# Inhibition of *Streptococcus mutans* Biofilm Formation and Virulence by *Lactobacillus plantarum* K41 Isolated From Traditional Sichuan Pickles

**DOI:** 10.3389/fmicb.2020.00774

**Published:** 2020-04-30

**Authors:** Guojian Zhang, Miao Lu, Rongmei Liu, Yuanyuan Tian, Viet Ha Vu, Yang Li, Bao Liu, Ariel Kushmaro, Yuqing Li, Qun Sun

**Affiliations:** ^1^Department of Food Science and Technology, College of Light Industry, Textile and Food Engineering, Sichuan University, Chengdu, China; ^2^State Key Laboratory of Oral Diseases, National Clinical Research Center for Oral Diseases, Department of Cariology and Endodonics, West China Hospital of Stomatology, Sichuan University, Chengdu, China; ^3^Key Laboratory of Bio-resources & Eco-environment of the Ministry of Education, College of Life Sciences, Sichuan University, Chengdu, China; ^4^Avram and Stella Goldstein-Goren Department of Biotechnology Engineering, Ben-Gurion University of the Negev, Beersheba, Israel

**Keywords:** *Streptococcus mutans*, *Lactobacillus plantarum*, sichuan pickles, antibacterial properties, dental caries

## Abstract

Among cariogenic microbes, *Streptococcus mutans* is considered a major etiological pathogen of dental caries. *Lactobacilli* strains have been promoted as possible probiotic agents against *S. mutans*, although the inhibitory effect of *Lactobacilli* on caries has not yet been properly addressed. The objective of this study was to screen *Lactobacillus* strains found in traditional Sichuan pickles and to evaluate their antagonistic properties against *S. mutans in vitro* and *in vivo*. In the current study, we analyzed 54 *Lactobacillus* strains isolated from pickles and found that strain *L. plantarum* K41 showed the highest inhibitory effect on *S. mutans* growth as well as on the formation of exopolysaccharides (EPS) and biofilm *in vitro*. Scanning electron microscopy (SEM) and confocal laser scanning microscope (CLSM) revealed the reduction of both EPS and of the network-like structure in *S. mutans* biofilm when these bacteria were co-cultured with strain *L. plantarum* K41. Furthermore, when rats were treated with strain *L. plantarum* K41, there was a significant reduction in the incidence and severity of dental caries. Due to K41’s origin in a high salinity environment, it showed a high tolerance to acids and salts. This may give this strain an advantage in harsh oral conditions. Results showed that *L. plantarum* K41 isolated from traditional Sichuan pickles effectively inhibited *S. mutans* biofilm formation and thus possesses a potential inhibitory effect on dental caries *in vivo*.

## Introduction

Dental caries is a common biofilm-dependent oral disease in humans, which manifests itself as a progressive demineralization of calcareous tissues caused by the complicated interactions between acid-generating bacteria and fermentable carbohydrates (Bal et al., 2019). The burden of untreated dental caries is shifting from children to adults, and caries in permanent teeth is one of the most prevalent disease world-over (Kassebaum et al., 2015). According to the 4th National Oral Health Survey of China 3-, 4-, and 5-years-old children, show the prevalence of dental caries of 50.8, 63.6, and 71.9%, respectively (Du et al., 2018). Dental biofilms are some of the most complex biofilm systems in nature. They are composed of multiple bacteria and fungi embedded in a matrix of polymers covering the surfaces of teeth (Xiao et al., 2012; Kidd and Fejerskov, 2016). Bacterial cells in the biofilm exhibit low metabolic activity, strong drug tolerance, and specific phenotype changes caused by the cell signaling or cross-species reciprocal protection (Yue et al., 2018). Previous studies have shown that *Streptococcus mutans* is an important oral cariogenic bacterium (He et al., 2013; Kulshrestha et al., 2016). Although it does not always dominate human dental plaque, in the presence of sucrose *S mutans* can assemble an insoluble exopolysaccharide (EPS). This EPS acts as a supportive framework for diffusion within the oral biofilm structure (Koo et al., 2013; Kim et al., 2015; Andre et al., 2017).

Currently, this cariogenic structure is eradicated mainly using non-specific mechanical removal such as tooth brushing and flossing, or by treatment with mouthwashes containing chlorhexidine (Gunsolley, 2010), essential oils (Van Leeuwen et al., 2011), or cetylpyridinium chloride (Haps et al., 2008). Fluoride washes are also used for the prevention of dental caries. In addition, natural substances such as tea catechins (Xu et al., 2012) and cranberry constituents (Koo et al., 2010), small molecules including dihydrofolate reductase (Zhang et al., 2015) and 7-epiclusianone (Murata et al., 2010), have also been characterized to show anti-plaque activities through unselective killing of oral microorganisms. However, few of them selectively eliminate cariogenic bacteria without disturbing the ecological balance of oral cavity. Therefore, novel therapies like probiotics (Kaye, 2017; Pahumunto et al., 2019) and glucansucrase inhibitors (Ito et al., 2011) have gained increasing attention for their antimicrobial activities in dental biofilm.

Probiotic microorganisms are living microbes that are beneficial to general health of hosts when taken in sufficient quantities. Delivery of probiotics to teeth as a paste or wash may concentrate the probiotic bacteria in the region of dental biofilm and thus may eradicate or diminish more pathogenic bacteria (Bosch et al., 2012; Fernandes et al., 2018). The most common strains found in commercial dental probiotic products include the genera *Lactobacillus* and *Bifidobacterium* (Sanders and Marco, 2010). However, both *in vitro* and clinical investigations of their effectiveness yielded ambiguous results with regards to their effects on *Lactobacillus* strains associated with caries (Fitzgerald et al., 1966; Tahmourespour and Kermanshahi, 2011; Hasslöf et al., 2013; Campus et al., 2014; Lin and Pan, 2014; Rodríguez et al., 2016).

*Lactobacillus plantarum* has been widely used in the preservation of cooked meat products (Vermeiren et al., 2004), condiments, and dairy products (Li et al., 2017). It exerts several beneficial effects, including immune system regulation, stabilization of the intestinal microbiota, and reducing cholesterol level (Vries et al., 2006). Furthermore, metabolic products of *L. plantarum*, including lactic acid and bacteriocin, have the antagonistic activities against adverse microorganisms (Zhu et al., 2014; Li et al., 2016). However, the inhibitory effect of *L. plantarum* on the control of caries has been only sparsely reported, and all the studies available to date were performed *in vitro* (Chun et al., 2013; Guo et al., 2016). Moreover, only a few studies have been performed on probiotics that can be added to daily foods directly. Therefore, an effort to control dental caries using probiotics is still necessary. Here, we propose exploring the anti-caries activity of *L. plantarum*, a bacterium that may be potentially supplemented in our daily diet.

Pickles are traditional fermented foods that are an integral part of the diet in the southwest of China due to their unique taste and beneficial functions. The numerous beneficial properties of pickles are conferred to them by the presence of microorganisms that contribute to the fermentation process. *L. plantarum* is one of the major contributors to these processes (Tian et al., 2013; Zhang et al., 2013). However, to date, no relevant reports have been published on the relationship between Sichuan pickles and oral health. We hypothesize that some *L. plantarum* strains in these pickles may be beneficial to the oral health by affecting the oral microbiome. We therefore undertook to evaluate the inhibitory effect of *L. plantarum* isolated from Sichuan pickles on dental caries both *in vitro* and *in vivo*.

## Materials and Methods

### Bacterial Strains and Growth Conditions

A total of 14 samples of pickles, the traditional fermented products in China, were collected from different areas in Sichuan province. All the samples were cut into pieces and subjected to serial dilution using sterile saline (0.80 g/100 mL), then suitable dilutions were spread on to the deMan-Rogosa-Sharpe (MRS) agar plates and incubated at 37°C for 48 h (Vijayendra et al., 2009). We preliminarily selected 54 mucoid colonies according to their morphological characteristics, as they were expected to be lactic acid bacteria, which were then stored at −80°C. According to the growth inhibition assay against *S. mutans*, five strains that exerted antibacterial activities were chosen. DNA of these five strains was extracted using QIAamp DNA Mini Kit following the manufacturer’s instruction and later subjected to polymerase chain reaction (PCR) using the universal primers of 27F and 1492R. According to their NCBI BLAST of 16S rDNA sequence, all these strains had the highest similarity to *L. plantarum*. The same results were obtained by matrix-assisted laser desorption ionization-time of flight mass spectrometry identification. The ABY-8 is a freeze-dried culture containing *Streptococcus thermophilic* and *Lactobacillus bulgaricus* used in yogurt fermentation.

All *Lactobacillus* strains and *S. thermophilic* were cultured in deMan-Rogosa-Sharpe (MRS) media. *S. mutans* UA159 was routinely grown in brain-heart infusion (BHI) medium (Difco, Detroit, MI, United States) in an anaerobic chamber (5% CO_2_, 10% H_2_, and 85% N_2_) at 37°C. All bacteria were stored at −80°C in 50% glycerol. After overnight incubation at 37°C, bacterial suspensions of both *S. mutans* and *Lactobacillus* were diluted by 10-fold using fresh media and cultured for approximately 2 h till reaching an OD_600_ = 0.5 before use.

### Growth Inhibition Assay

The antibacterial activities of *Lactobacillus* strains against *S. mutans* was quantified using a modified well-diffusion method (Lin and Pan, 2014). As shown in Supplementary Figure S1, 200 ml melted BHI agar medium held at about 45°C was inoculated with 20 μl well mixed bacterial suspension of *S. mutans*. The medium containing S. *mutans* was poured into plates quickly, then placed in Oxford cups (San Ai Si Scientific Instrument Co., Ltd., Yancheng, Jiangsu, China) for medium solidification. Oxford cups of 7.8 mm diameters were then filled with 200 μl bacterial suspension of *Lactobacillus* strains, or chlorhexidine acetate (0.02%, Madam Health pharmaceutical Co., Ltd., Shanghai, China) that was chosen as the positive control. The growth inhibition diameter was measured after incubation at 37°C for 24 h.

### Biofilm Formation Assay

The inhibitory effect of *Lactobacillus* sp. on *S. mutans* biofilm formation was performed as previously reported (Soderling et al., 2011). In brief, both *Lactobacilli* strains and *S. mutans* were diluted by 50-fold in the corresponding medium supplemented with 1% (w/v) sucrose and 25 μl MES (Sigma, St. Louis, MO, United States). Then, the formation of *S. mutans* biofilm was evaluated in the absence or presence of *Lactobacillus* strains. *Lactobacilli* strains were also cultivated alone to establish mono-species biofilms. Dilution of *S. mutans* and *Lactobacillus* strains were mixed at equal ratios (chlorhexidine acetate was used as the positive control). Biofilms were formed in 24-multiwell plates in order to enable viable cells measurements, and on sterile glass slides for microscopic observation.

In order to measure the viable cells in the biofilms, the unattached cells were removed from the wells after anaerobic incubation at 37°C for 18∼24 h. The remaining cells adhering to the wells were washed three times and resuspended in 1 ml PBS buffer. The re-suspensions were then collected and cultured in BHI agar plates supplemented with 0.1% (m/m) bromocresol. The CFU of *S. mutans* and *Lactobacillus* strains were enumerated following a culture period of 48 h (Schwendicke et al., 2017).

### Extracellular Polysaccharide Measurement

The “Anthrone-sulfuric acid method” was used to identify EPS production as previously described by Ren et al. (2016). After *Lactobacillus* strains and *S. mutans* were cultured to reach an OD_600_ = 0.5, *S. mutans* were diluted by 50-fold in BHI medium supplemented with 2% (w/v) sucrose. The bacterial suspensions of *Lactobacillus* strains were mixed with an equal volume of diluted *S. mutans* suspension. MRS medium was used as a control. After anaerobic incubation at 37°C for 24 h, the culture fluid was removed and replaced with 2 ml sterile PBS. The deposits were resuspended and washed three times with sterile PBS to remove the water-soluble EPS. Then, insoluble EPS was extracted using 1.0 M NaOH with agitation at 37°C for 2 h. EPS production was measured at the absorbance of 620 nm, and a standard curve was made using glucose.

### Autoaggregation and Coaggregation Assay

An autoaggregation assay was carried out as follows. *Lactobacillus* cells resuspended in PBS were adjusted to an absorbance of 0.6 ± 0.02 at A_600_ (A_0_); the absorbance (A_t_) of the upper suspension was measured in the following 4 h. The result was calculated as: Autoaggregation (%) = 1 – (A_t__/_A_0_) × 100, where A_t_ represents the absorbance at time *t* = 1, 2, 3 or 4 h.

For the coaggregation assay, equal volumes of *Lactobacillus* sp. and *S. mutans* were mixed, and the absorbance of the upper suspension was measured in the following 4 h. Coaggregation was calculated as follows: Coaggregation (%) = (((A_0_ + B_0_)/2) – C_t_)/((A_0_ + B_0_)/2) × 100, where A_0_ and B_0_ represent the initial absorbance of *Lactobacillus* sp. and *S. mutans*, and C_t_ represents the absorbance of the mixture measured at time *t* = 1, 2, 3 or 4 h.

### Tolerance to Acids and Salts

To test the acid tolerance, *Lactobacillus* sp. was inoculated in MRS medium with a pH of 3∼6 in the treated group, and pH 7 in the control group. The salt tolerance was evaluated in MRS medium supplemented with NaCl 0.5%, 1.0%, 2.0%, 4.0%, and 8.0%, while the untreated MRS medium represented the control group, after incubation of *Lactobacillus* sp. at 37°C for 24 h. The survival rate was calculated as follows: survival rate (%) = treated group/control group × 100.

### Minimum Inhibitory Concentration to Antibiotics

The minimum inhibitory concentration (MIC) of K41 against antibiotics was determined by the broth micro-dilution method using the standardized lactic acid bacteria susceptibility test medium (LSM), and *L. plantarum* ATCC 14917T was used as control (International Organization of Standardization/International Dairy Federation (ISO10932/Idf 223), 2010). The antibiotics concentrations in the plates were shown in Supplementary Table S1. The MIC was defined as the lowest concentration of the antibiotics that completely inhibited the growth of the tested strain. Each test was performed in triplicate.

### Scanning Electron Microscope Observation

Scanning electron microscopy (SEM) observation was performed as previously described (Wu et al., 2015). Sterile glass slides were added to a 24-multiwell plate. Biofilms were grown on slides and incubated under anaerobic conditions at 37°C for 24 h. Glass slides were gently washed with PBS buffer twice and fixed with 2.5% glutaraldehyde overnight, then dehydrated in graded ethanol solutions (30%, 40%, 50%, 60%, 70%, 80%, 85%, 90%, 95%, 100%) for 15 min, dried in liquid CO_2_, and sputter-coated with gold before observation (FEI, Hillsboro, OR, United States). SE mode was used for SEM evaluation, and the scanning parameter was set at 20.00 kV.

### Confocal Laser Scanning Microscope Observation

Confocal laser scanning microscope (CLSM) observation was performed as previously described with some modification (Cheng et al., 2016). All biofilms were dyed with Alexa Fluor 647-labeled dextran conjugate (1 μM; Life Technologies, Grand Island, NY, United States) and cultured on glass slides away from light for 24 h. Glasses were washed twice and labeled with 40 μL SYTO 9 green fluorescent nucleic acid stain (2.5 μM; Life Technologies, Grand Island, NY, United States) for 15 min. Then glass slides with biofilms were examined using a Leica DMIRE2 CLSM (Leica, Wetzlar, Germany) under a 60 × oil immersion objective lens. Emission wavelength at 668 nm was used for the detection of the EPS stained by Fluor 647-labeled dextran conjugate, while 498 nm was used for bacteria stained by SYTO 9 green fluorescent nucleic acid stain. Each image series was generated using optical sectioning at each position. Three-dimensional reconstruction and quantification of EPS/bacteria biomass at each position of biofilm were performed using IMARIS 7.0.0 (Bitplane, Zurich, Switzerland).

### Animals and Diet

The dosing experiments were performed on 28, 17-day-old females of specific-pathogen-free Sprague Dawley rats (Dashuo Inc., Chengdu, China), weighing 60 ± 5 g. The flow chart of the animal model is shown in Figure 4A. During the first 3 days, the rats were fed with a diet supplemented with 0.1% carbenicillin, 0.1% chloramphenicol, and water containing 4,000 U/ml penicillin. From the fifth day on, rats were fed with a diet no. 2000 (56% sucrose; Dashuo Inc.), and 10% sucrose was added into the drinking water with *ad libitum* access (Murata et al., 2010). At the age of 21 days, rats were infected with an overnight bacterial suspension of *S. mutans* UA159 (10^8^ CFU/ml) administered orally for three consecutive days. At age 24 days, the infection was assessed, and rats were randomly divided into four groups (*n* = 8): group 1, Control (MRS medium); group 2, Chlorhexidine acetate (0.02%); group 3, K41 (10^8^ CFU/ml); group 4, K41 alone (10^8^CFU/ml) mixed with ABY-8 (10^8^ CFU/ml). Rat-molars were topical treated daily as mentioned above using oral swabs at a unified time of 20 s per quadrant for a period of 35 days (Beiraghi et al., 1990). Rats were weighed twice a week for the first 2 weeks and then once a week. After 5 weeks of treatment, the animals were sacrificed, and the maxillae and mandibles were aseptically removed. This study was reviewed and approved by the Ethics Committee of the College of Life Sciences, Sichuan University (No. 20191217001; Sichuan University, Chengdu, China).

### Caries Scoring Assay

After flesh removal from around each tooth, the teeth were stained with 0.4% murexide solution for 12 h, then rinsed with distilled water (Han et al., 2017). Maxillary and mandibular molars were ground up along the mesiodistal sagittal plane using the rainbow technique (Super-Snap kit). Molar caries was evaluated and scored according to the Keyes’ method using a stereoscopic microscope (Keyes, 1958). Caries scoring was established by two expert examiners, who carried out blind scorings as the jaws were mixed-up and randomly assigned to the examiners.

### Micro-CT Analysis

The right halves of the maxilla of five rats in each group were scanned at 10 μM isotropic voxel resolution using a cone-beam Micro-CT system (SCANCO MEDICAL, Swiss). The scanning parameters were set at 70 kV and 200 μA. After scanning, three-dimensional images of the maxilla were reconstructed, and the enamel volume and mineral density of the first molars were analyzed.

### Laser Fluorescence Intensity Assessment

DIAGNOdent pen, a new generation of laser fluorescence device, was used for caries detection and quantification of the left halves of maxilla and mandible of eight rats in each group. Smooth surfaces and occlusal surfaces of each tooth were scored using a DIAGNOdent pen (model 2190) fitted with a fissure probe (Mat. no. 1.002.6967, KaVo, Biberach, Germany) and a proximal probe (Mat. no. 1.002.6970, KaVo, Biberach, Germany) that emitted visible red laser at a wavelength of 655 nm. The DIAGNOdent pen was calibrated according to the manufacturer’s guidelines with a ceramic standard (Rams and Alwaqyan, 2017). In all cases, the peak value was recorded as the final data for the surface. Every jaw had eight smooth surfaces and three occlusal surfaces to be assessed. These resulted in 112 smooth surfaces and 42 occlusal surfaces assessed in total. The readings of the DIAGNOdent pen in the smooth surfaces were the following: 0∼7, healthy tooth substance; 8∼15, beginning of the demineralization; ≥15, strong demineralization. The readings in the occlusal surfaces were the following: 0∼12, healthy tooth substance; 13∼24, beginning of the demineralization; ≥25, strong demineralization.

### Statistical Analysis

Statistical analysis was performed using SPSS Statistics 17.0. Differences among groups were evaluated using one-way analysis of variance (ANOVA) with Duncan’s multiple comparison test. Values are expressed as mean ± standard deviation (SD). A value of *P* < 0.05 was considered statistically significant.

## Results

### Inhibitory Effect of *Lactobacillus* sp. on *S. mutan*s Growth, Biofilm Formation, and EPS Production

A total of 54 *Lactobacillus* strains isolated from pickles were used to analyze their inhibitory effect on *S. mutans* growth. Six of the strains formed an inhibitory area around *S. mutans* (Table 1). According to the diameter of the inhibition area, the highest antimicrobial activity, 14.7 ± 1.5 mm, was observed for strain K41. This was slightly higher than the chlorhexidine acetate (14.2 ± 1.9 mm), indicating that K41 had the best inhibitory effect on the growth of *S. mutans*.

**TABLE 1:** Effect of *Lactobacillus* strains on the growth of *Streptococcus mutans*.

**Strains**	**Inhibition diameter (mm)**	**Strains**	**Inhibition diameter (mm)**	**Strains**	**Inhibition diameter (mm)**
Control^*a*^	ND^*b*^	K36	ND	K91	ND
Chlorhexidine acetate (0.02%)	14.2 ± 1.9^*Ac*^	K41	14.7 ± 1.5^A^	K92	ND
K11	11.3 ± 4.9^*A*^	K42	ND	K93	ND
K12	ND	K43	ND	K101	ND
K13	ND	K44	ND	K102	ND
K14	ND	K45	ND	K103	ND
K15	ND	K46	ND	K111	ND
K16	ND	K51	ND	K112	ND
K21	ND	K52	ND	K113	ND
K22	ND	K53	8.8 ± 1.0^*B*^	K121	ND
K23	ND	K61	ND	K122	ND
K24	ND	K62	ND	K123	ND
K25	ND	K63	ND	K131	ND
K26	10.3 ± 1.5^*A*^	K71	ND	K132	ND
K31	ND	K72	ND	K133	ND
K32	ND	K73	ND	K141	ND
K33	ND	K81	ND	K142	11.3 ± 1.2^*A*^
K34	13.3 ± 1.0^*A*^	K82	ND	K143	ND
K35	ND	K83	ND		

*^*a*^Control: deMan-Rogosa-Sharpe media. ^*b*^ND: not detectable. ^*c*^Values are expressed as mean ± SD of the inhibition diameter (mm). Results with different superscript capital letters are significantly different (*P* < 0.05).*

**FIGURE 1:**
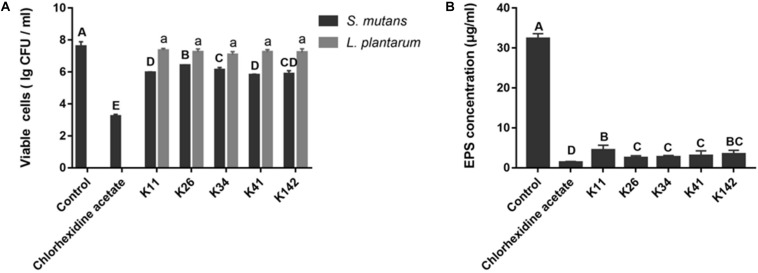
Screening of potential probiotics with antagonistic properties against *S. mutans*. **(A)** Viable cells of *S. mutans* and *Lactobacillus* sp. in different biofilms. **(B)** EPS production by *S. mutans* in the presence of a bacterial suspension of *Lactobacillus* strains. Control: deMan-Rogosa-Sharpe media. Results with different letters are significantly different (*P* < 0.05).

The inhibitory effect on *S. mutans* biofilm formation is illustrated in Figure 1A. Viable *S. mutans* cells in the chlorhexidine acetate group were significantly different from the control group (*P* < 0.05). *L. plantarum* K11 and K41 possessed a similar magnitude of bactericidal activity against *S. mutans*, their inhibition rates were 97.7 and 98.4%, respectively. Interestingly, the viability of the different *Lactobacillus* sp. in the biofilm was not significantly different (*P* > 0.05).

As shown in Figure 1B and Supplementary Figure S4, *L. plantarum* K26, K34 and K41, showed no significant differences with regards to EPS concentration. Furthermore, K41 showed a better inhibitory effect on EPS formation than K11 (*P* < 0.05). Therefore, *L. plantarum* K41 should be considered the strain with the best inhibitory effect on *S. mutans* growth, biofilm formation and EPS production, of *Lactobacillus* sp. perhaps indicating its potential benefit on the control of dental caries.

### The Aggregation Ability and Tolerance of *Lactobacillus* sp. to Oral Condition

**FIGURE 2:**
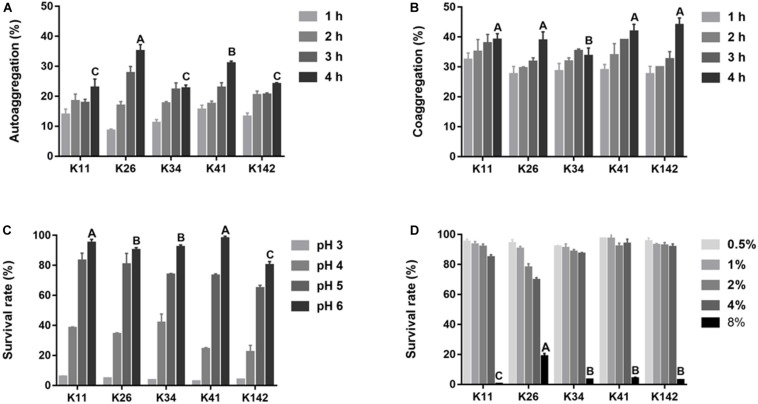
Aggregation ability and tolerance to harsh oral condition of *L. plantarum*. **(A)** The ability of autoaggregation measured at 1, 2, 3, and 4 h. **(B)** Coaggregation of *Lactobacillus* sp. with *S. mutan*s at 1, 2, 3, and 4 h. **(C)** *Lactobacillus* sp. survival rate in acid condition. **(D)** *Lactobacillus* sp. survival rate in salty condition. Control: deMan-Rogosa-Sharpe media. Values are expressed as the mean ± S.D. Results with different capital letters are significantly different (*P* < 0.05).

The autoaggregation ability of *Lactobacillus* is shown in Figure 2A and shows a significant difference among the five strains at 4 h. The best autoaggregation ability was K26 (35.1%), followed by K41 (31.0%). There was no significant difference in the remaining three strains (*P* > 0.05). The coaggregation with *S. mutans* increased during the first 4 h, and the difference among the five strains was small varying from 33.6 to 44.0% at 4 h (Figure 2B).

The survival rates of all strains were over 75% when the pH was 5∼6, but lower than 5% at pH 3 (Figure 2C). When the salinity was less than 4.0%, the survival rates of all five strains were greater than 70% (Figure 2D). According to the aggregation results and the tolerance to acids and salt, all tested strains had strong ability to survive in the harsh oral environment. Thus, due to its extensive bactericidal activity, *L. plantarum* K41 was chosen for the subsequent experiments.

The MIC distributions and breakpoints of K41 are presented in Supplementary Table S2. *L. plantarum* K41 was characterized as sensitive according to breakpoints proposed by the Clinical and Laboratory Standards Institute [CLSI] (2015) and European Food Safety Authority [EFSA] (2012).

### Inhibitory Effect of *L. plantarum* K41 on *S. mutans* Biofilm Structure

**FIGURE 3:**
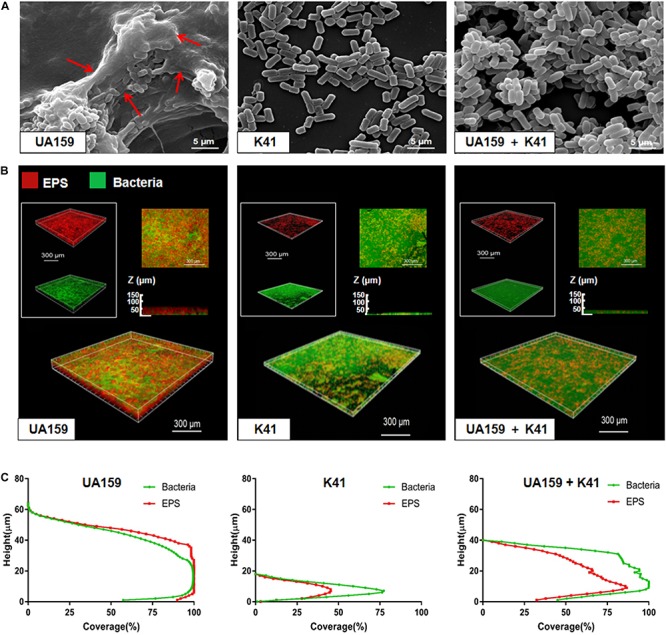
Microscope imaging of *L. plantarum* K41 on the *S. mutans* biofilm formation. **(A)** Scanning electron microscopy (SEM) images of the structures of different biofilms. Images were taken at 20,000× magnification. **(B)** EPS (red) and bacteria (green) distribution in the double-labeled biofilm was observed by confocal laser scanning microscope (CLSM). **(C)** The distribution of bacteria and EPS at different heights. The three-dimensional reconstruction was performed by IMARIS 7.0.0. Images were taken at 60× magnification. Red arrows in Figure 3A were the network-like structures.

Once it was determined at what point K41 exerted the best inhibitory effect on *S. mutans* biofilm formation, we investigated the structure of the biofilm when *S. mutans* was co-cultured with K41. The SEM micrograph in Figure 3A showed that UA159-species formed a compact biofilm covered by network-like structures, which were identified as EPS. The structure in UA159 biofilm, following the addition of K41, showed a looser biofilm when compared to UA159-species alone, and the amount of EPS was decreased. Moreover, K41-species biofilm showed the thinnest structure, and had few micro-colonies on the surface. CLSM results in Figure 3B and Supplementary Figure S2A confirmed that EPS in UA159 + K41 biofilm was less dense than UA159-species biofilm. Moreover, the biofilm formed by *S. mutans* UA159 was significantly thicker (65.0 μm) than that formed by *L. plantarum* K41 (18.0 μm) and by the mixed co-culture (40 μm) (Figure 3C). As shown in Supplementary Figure S2B, the EPS/bacterial ratio in UA159-species biofilm was greater than in UA159 + K41 biofilm, suggesting that *L. plantarum* K41 exerted an inhibitory effect on EPS formation in biofilm.

### Inhibitory Effect of *L. plantarum* K41on *S. mutans* Virulence *in vivo*

**FIGURE 4:**
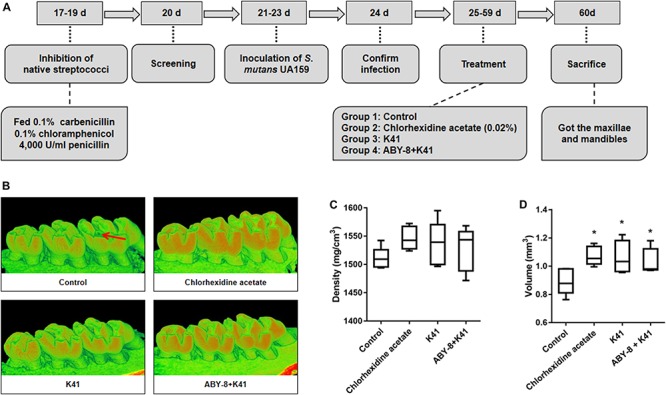
The observation and enamel analysis of maxillary by Micro-CT. **(A)** Flow chart of animal model. **(B)** Three-dimensional images of the molar area (right column) of the left mandible. **(C)** Density of the first molar enamel. **(D)** Volume of the first molar enamel. Control: deMan-Rogosa-Sharpe media; ABY-8: a mixture culture of *S. thermophilic* and *L. bulgaricus*. “*”: significant difference compared with control group (*P* < 0.05). Red arrows in Figure 4B were for tooth cavity. The red and green represented the degree of the mineralization, and the redder color means the higher degree of mineralization.

The rats remained in stable health throughout the entire experimental period. No significant weight gain or loss was observed in the treated groups (Supplementary Figure S3). Significant caries lesions were observed in all the untreated stained molars under stereoscopic microscopy (Table 2). Treatment with K41 significantly reduced the incidence and severity of smooth and sulcal caries compared with the negative control group (*P* < 0.05). The maxilla was reconstructed, and the three-dimensional images were performed in Figure 4B. The mean mineral density of the first molar enamel treated with K41 was higher than the one in the control group and lower than that in the positive control group (Figure 4C). In addition, the mean volume of the first molar enamel treated with K41 was larger than that of the molar treated with MRS medium and smaller compared to that of the molar treated with chlorhexidine acetate (*P* < 0.05) (Figure 4D). The condition of the smooth surfaces and occlusal surfaces of every jaw was recorded. The results are summarized in Table 3. The demineralization degree of the control group was more severe than the other three groups on both smooth and occlusal surfaces (*P* < 0.01), but no statistical difference was observed among chlorhexidine acetate (0.02%), K41 and K41 + ABY-8 group (*P* > 0.01).

**TABLE 2:** Effect of different treatments on the development of dental caries in rats (Keyes score).

**Treatment**	**Smooth-surface caries**	**Sulcal-surface caries**	**Sulcal-surface severity (mean ± SD)**		
			**Ds^*a*^**	**Dm^*b*^**	**Dx^*c*^**
Control^*d*^	55.4 ± 3.7^*Af*^	30.0 ± 3.6^*A*^	21.0 ± 2.5^*A*^	11.1 ± 3.4^*A*^	ND^*e*^
Chlorhexidine acetate (0.02%)	45.9 ± 3.4^*B*^	22.3 ± 2.3^*B*^	11.7 ± 2.4^*B*^	5.0 ± 2.6^*B*^	ND
K41	44.4 ± 4.6^*B*^	21.4 ± 4.1^*B*^	11.7 ± 3.5^*B*^	5.0 ± 1.3^*B*^	ND

*^*a*^Ds: dentin exposed. ^*b*^Dm: 3/4 of the dentin affected. ^*c*^Dx: whole dentin affected. ^*d*^Control: deMan-Rogosa-Sharpe media. ^*e*^ND: not detectable. ^*f*^Values followed by the same capital letter are not significantly different from each other.*

**TABLE 3:** DIAGNOdent laser autofluorescence intensity on rat teeth.

**Treatment**	**Smooth surface**					**Occlusal surface**				
	**Total^*a*^**	**H^*b*^**	**B^*c*^**	**S^*d*^**	**Mean Rank^*e*^**	**Total**	**H**	**B**	**S**	**Mean Rank**
Control^*f*^	112	58	46	8	269.92^*A*^	42	11	19	12	105.60^*A*^
Chlorhexidine acetate (0.02%)	112	83	29	0	217.06^*B*^	42	24	18	0	68.07^*B*^
K41	112	88	20	4	209.66^*B*^	42	19	22	1	78.29^*B*^
K41 + ABY-8	112	92	17	3	201.36^*B*^	42	16	23	3	86.05^*B*^

*^*a*^Total surface of each group. ^*b*^Healthy tooth substance, read as 0∼7 in smooth surfaces and 0∼12 in occlusal surfaces. ^*c*^Beginning of the demineralization, read as 8∼15 in smooth surfaces and 13∼24 in occlusal surfaces. ^*d*^Strong demineralization, read as ≥15 in smooth surfaces and ≥25 in occlusal surfaces. ^*e*^Statistically significant capital differences (*P* < 0.05) were observed between groups assessed. ^*f*^Control: deMan-Rogosa-Sharpe media.*

## Discussion

Dental plaque biofilm is the ecological structure formed by a variety of microorganisms deposited on the tooth surface, and it is a key process leading to dental caries (Matsumoto et al., 2010). With the frequent exposure to dietary carbohydrate, the microbial community of plaque biofilm microbial population gradually shifts toward to cariogenic bacteria with characteristics of acidogenicity and acidurity (Bowen and Koo, 2011). Extensive investigations showed that *S. mutans* was the primary causative agent specifically found in the dental plaque (Aida et al., 2018; Jeong et al., 2018). Many probiotic solutions purportedly affecting *S. mutans* have been reported to treat dental caries (Soderling et al., 2011; Gruner et al., 2016; Schwendicke et al., 2017). Indeed in recent years, probiotic products such as probiotic powders or yogurts are becoming more and more popular as treatments for caries (Nadelman et al., 2017). Thus, it is possible that the daily application of probiotic microorganisms like *Lactobacillus* sp. through the consumption of fermented products will provide an important novel probiotic treatment for dental caries.

Although numerous studies have explored the potential benefit of probiotics to oral health (Campus et al., 2014; Lin and Pan, 2014), there is currently no standardized or comprehensive protocol for screening novel oral probiotics *in vitro*. Although it has been reported that probiotics inhibiting the growth of *S. mutans* it was thought that it did not necessarily control dental caries. Despite this the inhibition of biofilm formation by probiotics may well be relevant in the reduction of carinogenicity (Schwendicke et al., 2017). The main action of *Lactobacillus* sp. observed here was the inhibition of the formation of *S. mutans* biofilm and the reduction of the dental caries occurrence. In our study, the inhibitory rate of biofilm formation by *S. mutans* when co-cultured with *L. plantarum* K41 was 98.4%, which was similar to that of *W. cibaria CMU* of approximately 95% (Jang et al., 2016). Moreover, the inhibitory effect of *L. plantarum* K41 against *S. mutans* was higher than chlorhexidine acetate. In addition, the inhibitory rate in the formation of insoluble EPS by K41 was 90.7%, which was significantly higher than that for the previously reported *L. plantarum* K25 (21.44%) (Guo et al., 2016). Therefore, *L. plantarum* K41 provides an inhibitory effect on biofilm formation, indicating its potential beneficial effect in control of dental caries.

*Lactobacillus* sp. may provide a beneficial effect of reducing the occurrence of dental caries by having an inhibitory effect on biofilm formation *in vitro*. To be effective it must also be able to survive in the condition of the mouth (Jeong et al., 2018). Indeed, it is possible that *Lactobacillus paracasei* F19 had no long-term effect on the incidence of caries due to poor viability under the oral conditions over a long time (Hasslöf et al., 2013). Adhesion to epithelial cells and mucosal surfaces is the primary indicator of the effect of *Lactobacillus* sp. in maintaining oral health, and it is a multistep process involving the composition, structure and forces of interaction related to the intestinal epithelial cells or mucosal surfaces (Hagerman et al., 2010). In most cases, autoaggregation is beneficial for adhesion, and coaggregation with *S. mutans* may form a barrier to prevent the action of pathogenic microorganisms (Del et al., 2000; Lang et al., 2010). In our research, the coaggregation and autoaggregation rates at 4 h of *L. plantarum* K41 were 41.87 and 31.01%, respectively, which was similar to *L. plantarum* K25 as previously reported (Guo et al., 2016). Moreover, as harsh oral conditions due to the intake of low-acid or high-salt food, may affect the viability of probiotics the ability of K41 to withstand these conditions make it a candidate for probiotics. In view of horizontal gene transfer occurring in dental biofilms, the microbiological breakpoints of Clinical Laboratory Standards Institute [CLSI] (2015) and European Food Safety Authority [EFSA] (2012) indicate that the use of K41 in food is safe. Moreover, it has been reported that the antibiotics’ resistance of the *Lactobacillus* strains used widely in fermentation processes is not likely to have the potential health threat to humans (Ma et al., 2017).

To further verify the inhibitory effect of *L. plantarum* K41 on dental caries, an animal model was established to examine whether K41 could reduce the incidence of caries. In a previous study, *L. paracasei* subsp. paracasei NTU 101 was found to be protective against the development of dental caries in rats (Lin and Pan, 2014), but no report is available to date with regards to the inhibitory effect of *L. plantarum* on dental caries in animal models. When compared with micro-CT, the process of laser fluorescence intensity assessment was simple and convenient and thus used here. This enabled the use of one half of the maxilla of five rats in each group for micro-CT analysis, while the other half and mandible of eight rats were used for the assessment of the degree of demineralization by laser fluorescence intensity assessment. According to the analysis of micro-CT, the density was not significantly different among the four groups (*P* > 0.05), perhaps due to the individual differences and sampling capacity. Moreover, caries scoring assay, which is widely used in the clinical examination of dental caries, was also used to detect the caries lesions. In our study, we found that caries lesions and demineralization degree in K41 group were significantly lower than those of control group, suggesting that K41 had also beneficial effect on the control of dental caries.

Recently, an increased interest in the application of probiotics for oral health has emerged, and the concept of using probiotics to prevent caries has been proposed (Isabelle and Teughels, 2015; Pahumunto et al., 2019). To date, probiotics have been added to some functional beverages, while fermented milk is considered to be the most widely food vehicle supplemented with probiotic bacteria due to its unique taste and high nutritional properties (Balthazar et al., 2017; Nadelman et al., 2017; Nadelman et al., 2019). Therefore, *L. plantarum* K41 shows a potential commercial value when added to dairy products.

However, the animal assay still has its limitations. Our animal assay missed evaluating the influence of *L. plantarum* K41 on lesions in dentin and root caries. Rat-molars were treated by *L. plantarum* K41 following *S. mutans* infection and were assessed, even if there was no gross morphological evidence of dental caries lesions. The colonization of the oral cavity by *Lactobacilli* requires a retentive niche (Caufield et al., 2005). Our animal assay could not prove the relationship of K41 with root caries. It was commonly accepted that *L. acidophilus* was dominant in deep caries samples (Martin et al., 2002). In the future, the effect of K41 on root or dentin caries should be fully investigated.

In conclusion, our work demonstrated that *L. plantarum* K41 isolated from traditional Sichuan pickles had an inhibitory effect on the biofilm formation of *S. mutans*. Our results offer a potential alternative strategy for the control of oral biofilm/dental plaque and dental caries.

## Data Availability Statement

All datasets generated for this study are included in the article/Supplementary Material.

## Ethics Statement

This study was carried out in accordance with the recommendations of the Swiss Animal Protection Ordinance. It was reviewed and approved by the Ethics Committee of the College of Life Sciences, Sichuan University (No. 20191217001; Sichuan University, Chengdu, China).

## Author Contributions

QS, YQL, GZ, and ML designed the studies. GZ and ML performed the experiments and wrote the manuscript. YT, RL, YL, and VV assisted the growth inhibition and biofilm formation experiments. RL, YL, and BL assisted the establishment of caries models. GZ, ML, YT, VV, AK, and BL analyzed the data. AK, QS, and YQL revised the manuscript. The manuscript has been reviewed and approved by all authors before submission.

## Conflict of Interest

The authors declare that the research was conducted in the absence of any commercial or financial relationships that could be construed as a potential conflict of interest.

## Supplementary Material

FIGURE S1 The growth inhibition assay plate.

FIGURE S2 Quantitative analysis in the double-labeled biofilm observed by confocal microscopy. **(A)** Quantification of bacteria and EPS biomass. **(B)** The ratio of EPS to bacteria at different heights in biofilms. Values are expressed as mean ± SD.

FIGURE S3 Effects of different treatments on rat weight gain. Values are expressed as mean ± S.D.

FIGURE S4 Standard curve for EPS quantitative determination by anthrone-sulfuric method.

TABLE S1 The concentration of antibiotics tested.

TABLE S2 The MIC distributions and breakpoints of *L. plantarum* against antibiotics tested.
