# Supplementary figures and images for: Inhibition of Streptococcus mutans Biofilm Formation and Virulence by Lactobacillus plantarum K41 Isolated From Traditional Sichuan Pickles
Source: Front Microbiol. 2020 Apr 30;11:774. doi: 10.3389/fmicb.2020.00774 (PMC7203412; doi:10.3389/fmicb.2020.00774)

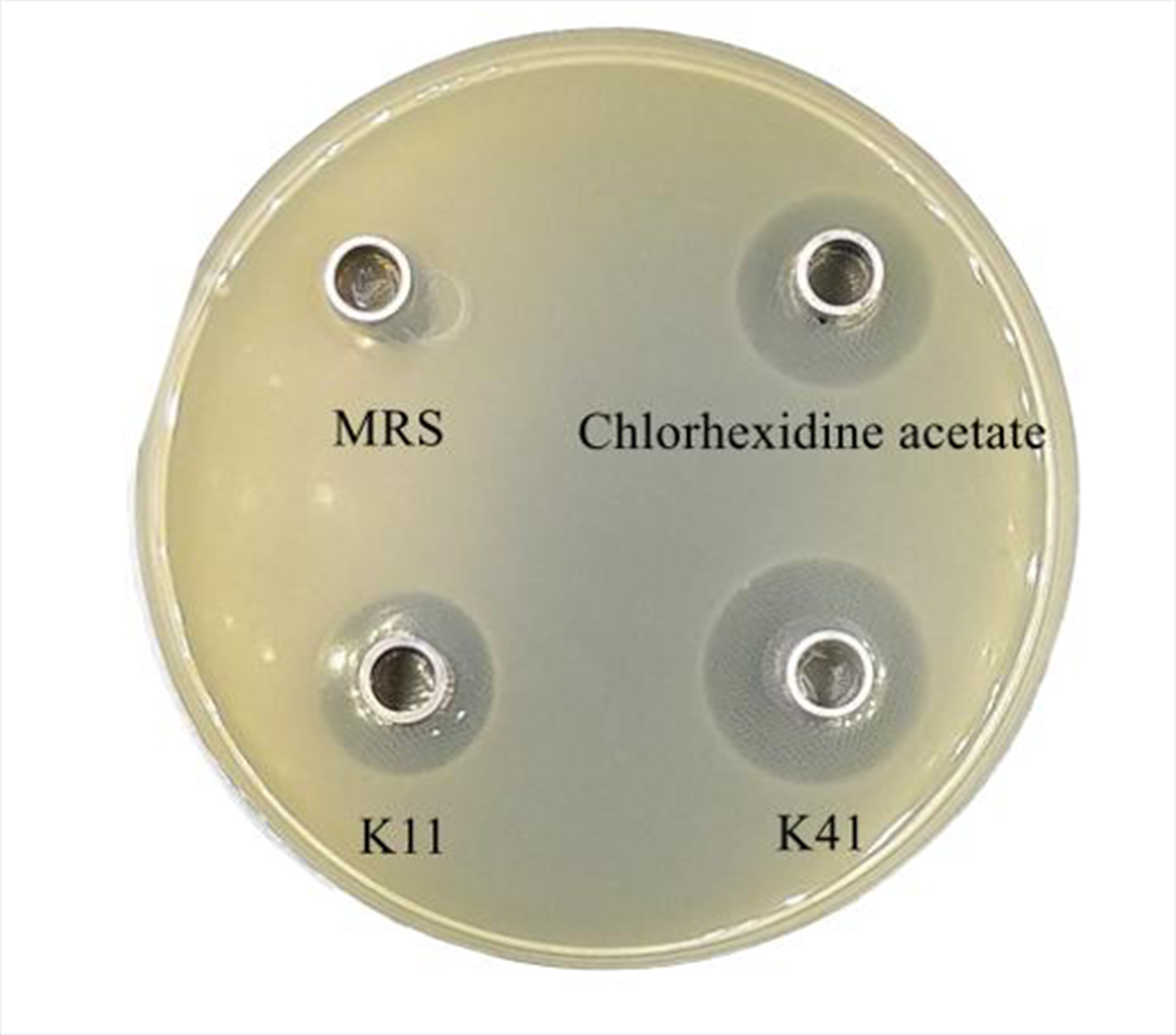

Supplement: FIGURE S1 — The growth inhibition assay plate. [file Image_1.TIF]

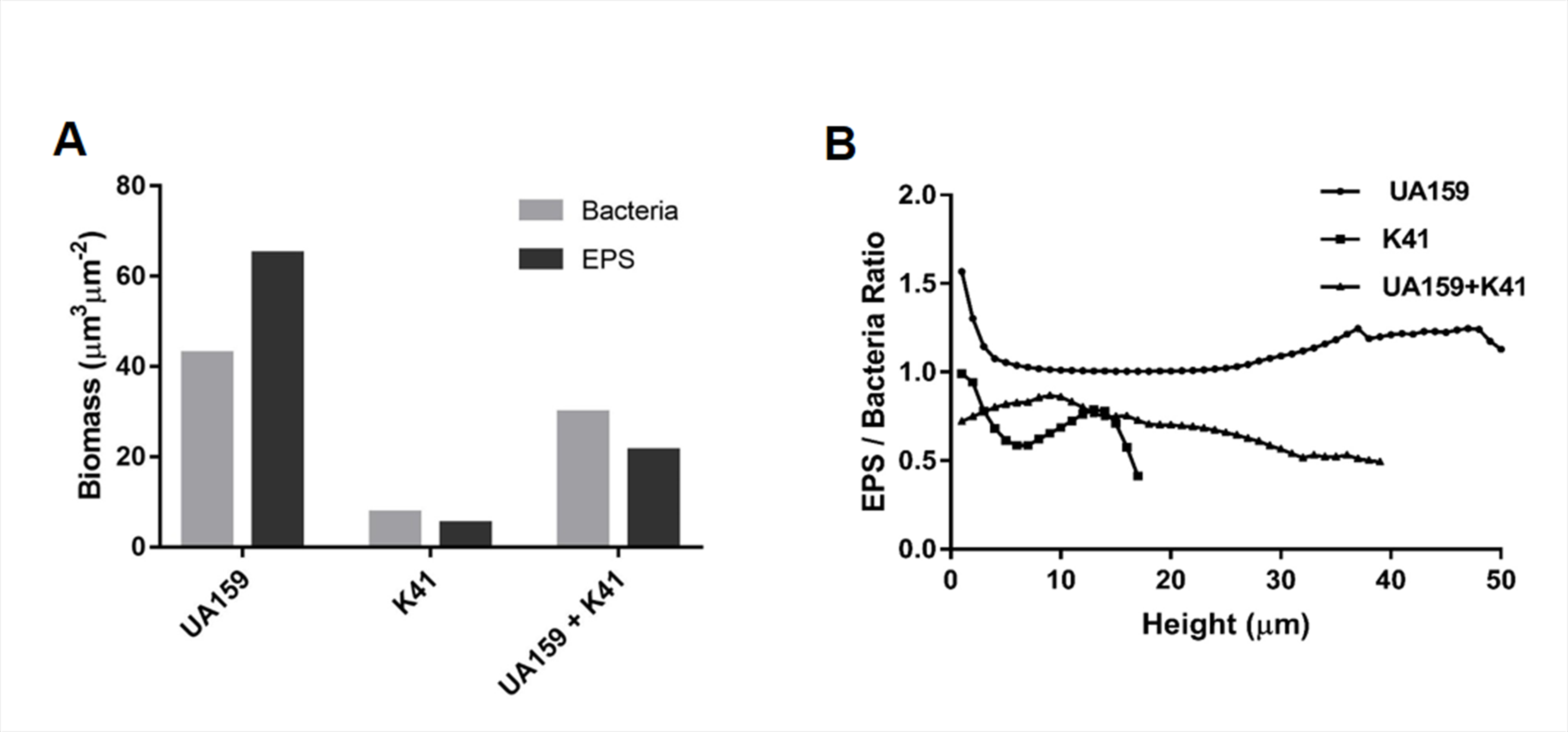

Supplement: FIGURE S2 — Quantitative analysis in the double-labeled biofilm observed by confocal microscopy. (A) Quantification of bacteria and EPS biomass. (B) The ratio of EPS to bacteria at different heights in biofilms. Values are expressed as mean ± SD. [file Image_2.TIF]

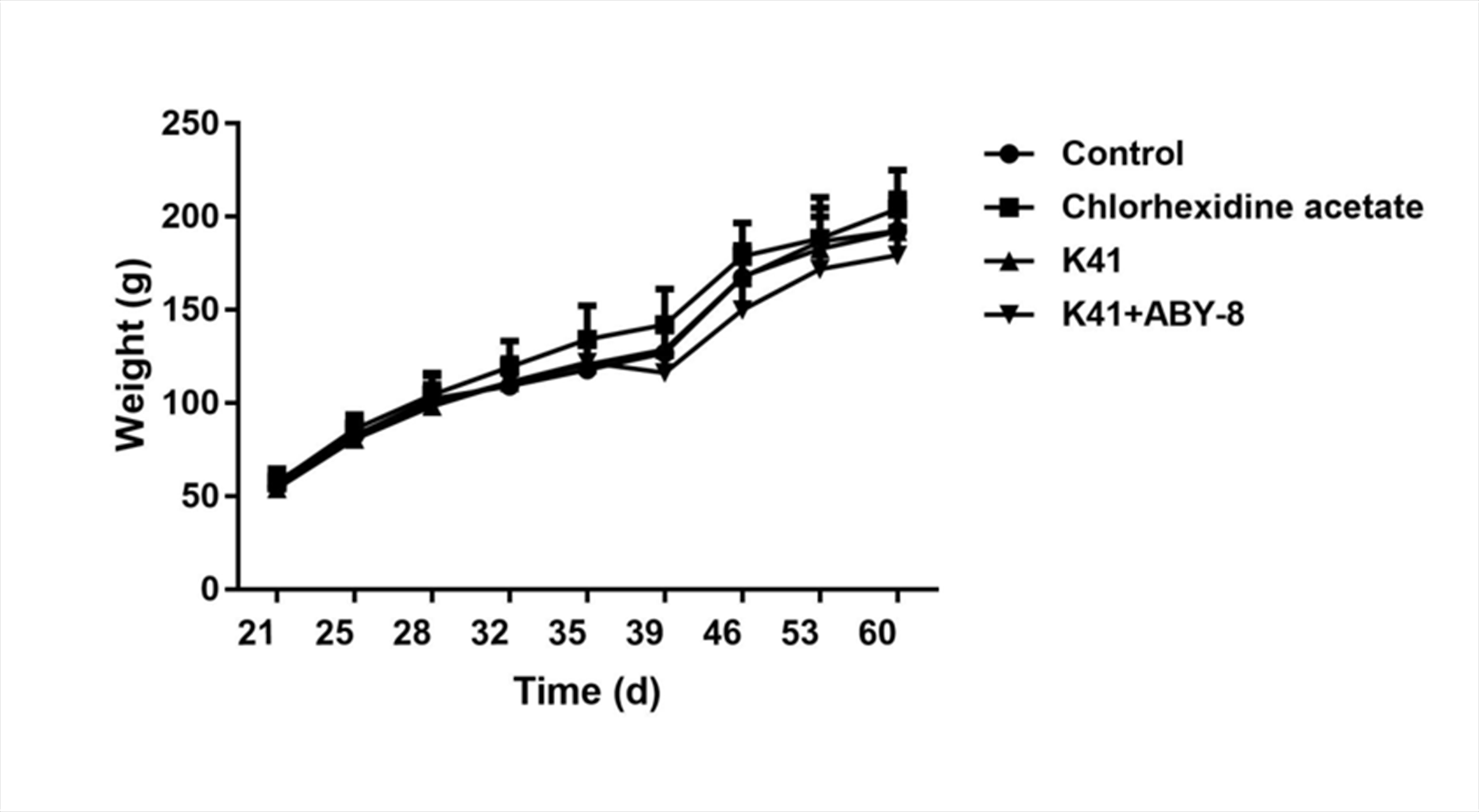

Supplement: FIGURE S3 — Effects of different treatments on rat weight gain. Values are expressed as mean ± S.D. [file Image_3.TIF]

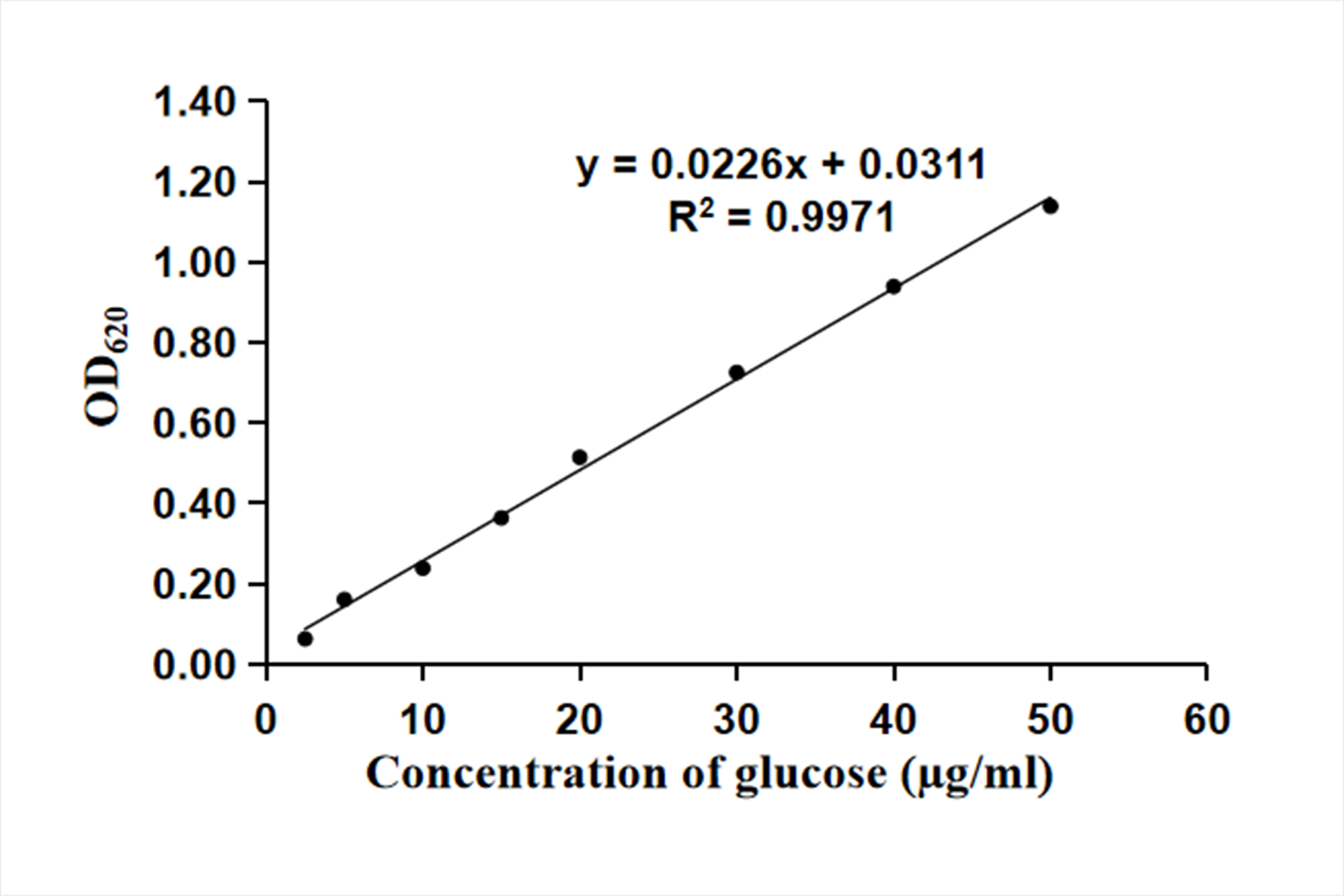

Supplement: FIGURE S4 — Standard curve for EPS quantitative determination by anthrone-sulfuric method. [file Image_4.TIF]
