# Supplementary material for: Inhibition of Streptococcus mutans Biofilm Formation and Virulence by Lactobacillus plantarum K41 Isolated From Traditional Sichuan Pickles
Source: Front Microbiol. 2020 Apr 30;11:774. doi: 10.3389/fmicb.2020.00774 (PMC7203412; doi:10.3389/fmicb.2020.00774)
Supplement: TABLE S1 — The concentration of antibiotics tested. [file Table_1.doc]

**Supplementary Table S1.** The concentration of antibiotics tested

| Antibiotics | Concentration (µg/mL) | | | | | | | | | |
| --- | --- | --- | --- | --- | --- | --- | --- | --- | --- | --- |
| Ampicillin | 0.032 | 0.063 | 0.125 | 0.25 | 0.5 | 1 | 2 | 4 | 8 | 16 |
| Penicillin | 0.032 | 0.064 | 0.125 | 0.25 | 0.5 | 1 | 2 | 4 | 8 | 16 |
| Imipenem | 0.016 | 0.032 | 0.064 | 0.125 | 0.25 | 0.5 | 1 | 2 | 4 | 8 |
| Meropenem | 0.016 | 0.032 | 0.064 | 0.125 | 0.25 | 0.5 | 1 | 2 | 4 | 8 |
| Erythromycin | 0.016 | 0.032 | 0.064 | 0.125 | 0.25 | 0.5 | 1 | 2 | 4 | 8 |
| Clindamycin | 0.032 | 0.063 | 0.125 | 0.25 | 0.5 | 1 | 2 | 4 | 8 | 16 |
| Linezolid | 0.032 | 0.063 | 0.125 | 0.25 | 0.5 | 1 | 2 | 4 | 8 | 16 |
| Tetracycline | 0.125 | 0.25 | 0.5 | 1.0 | 2 | 4 | 8 | 16 | 32 | 64 |
| Gentamycin | 0.5 | 1 | 2 | 4 | 8 | 16 | 32 | 64 | 128 | 256 |
