# Supplementary material for: Inhibition of Streptococcus mutans Biofilm Formation and Virulence by Lactobacillus plantarum K41 Isolated From Traditional Sichuan Pickles
Source: Front Microbiol. 2020 Apr 30;11:774. doi: 10.3389/fmicb.2020.00774 (PMC7203412; doi:10.3389/fmicb.2020.00774)
Supplement: TABLE S2 — The MIC distributions and breakpoints of L. plantarum against antibiotics tested. [file Table_2.doc]

**Supplementary Table S2.** The MIC distributions and breakpoints of *L. plantarum* against antibiotics tested.

| Antibiotics | The respective MIC values of *L. plantarum* (µg/mL) | | Microbiological breakpoints (µg/mL) |
| --- | --- | --- | --- |
|  | ATCC 14917T | K41 |  |
| Ampicillin^a^ | 0.064 | 0.064 | 8 |
| Penicillin^a^ | 0.5 | 1 | 8 |
| Imipenem^a^ | 0.032 | 0.032 | 0.5 |
| Meropenem^a^ | 0.064 | 0.064 | 1 |
| Erythromycin^a^ | 0.5 | 0.25 | 0.5 |
| Clindamycin^a^ | 0.125 | 0.125 | 0.5 |
| Linezolid^a^ | 2 | 1 | 4 |
| Tetracycline^b^ | 4 | 32 | 32 |
| Gentamycin^b^ | 2 | 1 | 16 |

The corresponding microbiological breakpoints were according to CLSI (2015)^a^ and EFSA (2012)^b^.
